# Early course of microcirculatory perfusion in eye and digestive tract during hypodynamic sepsis

**DOI:** 10.1186/cc11341

**Published:** 2012-05-15

**Authors:** Andrius Pranskunas, Vidas Pilvinis, Zilvinas Dambrauskas, Renata Rasimaviciute, Rita Planciuniene, Paulius Dobozinskas, Vincentas Veikutis, Dinas Vaitkaitis, E Christiaan Boerma

**Affiliations:** 1 Department of Intensive Care Medicine, Lithuanian University of Health Sciences, Eiveniu str. 2, LT-50009, Kaunas, Lithuania; 2 Department of Surgery, Lithuanian University of Health Sciences, Eiveniu str. 2, LT-50009, Kaunas, Lithuania; 3 Department of Microbiology, Lithuanian University of Health Sciences, Eiveniu 4, LT-50161, Kaunas, Lithuania; 4 Department of Disaster Medicine, Lithuanian University of Health Sciences, Eiveniu 4, LT-50161, Kaunas, Lithuania; 5 Institute of Cardiology, Lithuanian University of Health Sciences, Sukileliu 17, LT-50009, Kaunas, Lithuania; 6 Department of Intensive Care Medicine, Medical Center Leeuwarden, Henri Dunantweg 2, Leeuwarden, 8901 BR, The Netherlands; 7 Department of Translational Physiology, Academic Medical Center, Amsterdam, The Netherlands

## Abstract

**Introduction:**

The aim of the study was to evaluate and compare the microcirculatory perfusion during experimental sepsis in different potentially available parts of the body, such as sublingual mucosa, conjunctiva of the eye, and mucosa of jejunum and rectum.

**Methods:**

Pigs were randomly assigned to sepsis (*n *= 9) and sham (*n *= 4) groups. The sepsis group received a fixed dose of live *Escherichia coli *infusion over a 1-hour period (1.8 × 10^9^/kg colony-forming units). Animals were observed 5 hours after the start of *E. coli *infusion. In addition to systemic hemodynamic assessment, we performed conjunctival, sublingual, jejunal, and rectal evaluation of microcirculation by using Sidestream Dark Field (SDF) videomicroscopy at the same time points: at baseline, and at 3 and 5 hours after the start of live *E. coli *infusion. Assessment of microcirculatory parameters of convective oxygen transport (microvascular flow index (MFI) and proportion of perfused vessels (PPV)), and diffusion distance (perfused vessel density (PVD) and total vessel density (TVD)) was done by using a semiquantitative method.

**Results:**

Infusion of *E. coli *resulted in a hypodynamic state of sepsis associated with low cardiac output and increased systemic vascular resistance despite fluid administration. Significant decreases in MFI and PPV of small vessels were observed in sublingual, conjunctival, jejunal, and rectal locations 3 and 5 hours after the start of *E. coli *infusion in comparison with baseline variables. Correlation between sublingual and conjunctival (*r *= 0.80; *P *= 0.036), sublingual and jejunal (*r *= 0.80; *P *= 0.044), and sublingual and rectal (*r *= 0.79; *P *= 0.03) MFI was observed 3 hours after onset of sepsis. However, this strong correlation between the sublingual and other regions disappeared 5 hours after the start of *E. coli *infusion. Overall, the sublingual mucosa exhibited the most-pronounced alterations of microcirculatory flow in comparison with conjunctival, jejunal, and rectal microvasculature (*P *< 0.05).

**Conclusions:**

In this pig model, a time-dependent correlation exists between sublingual and microvascular beds during the course of a hypodynamic state of sepsis.

## Introduction

Sepsis is a systemic inflammatory response to infection, and the microcirculation is the primary site of alteration [1]. Sepsis is a highly dynamic, acute illness with a relatively high risk for progression to septic shock, organ failure, and death [2], despite adequate infection control and aggressive resuscitation [3]. In this setting, alterations in the microcirculation can still be present, even if the systemic hemodynamic is optimized [4,5]. In human studies using orthogonal polarization spectral (OPS) or sidestream dark-field (SDF) videomicroscopy, these alterations in the sublingual site can be observed very early, within a few hours of sepsis [6]. The microcirculation is evaluated mostly in a sublingual location. Studies show that sublingual microcirculatory alterations may reflect intestinal microcirculatory perfusion deficits in sepsis, albeit under specific circumstances [7]. However, it is of great interest to discover how sublingual microcirculation can reflect other beds of microcirculation, because some data demonstrate the heterogeneity of perfusion within and between organs during sepsis [5,8].

Conjunctival microcirculation is readily accessible and may be the closest window for evaluation of brain microcirculation in sepsis. Internal carotid artery stenosis was inversely correlated with the ipsilateral and contralateral decrease in conjunctival functional capillary density in nonsepsis patients [9]. No studies have investigated the conjunctival microcirculation and its correlation with microcirculatory changes in the sublingual location during sepsis.

The aim of our study was to evaluate the microcirculatory perfusion of potentially accessible parts of the body, such as the sublingual, conjunctival, jejunal, and rectal mucosa, at the same time points, and to establish their correlation over time during experimental sepsis.

## Material and methods

Animals were treated by following the National Institutes of Health guidelines and directions for the care and use of experimental animals in our institution. The study protocol was approved by the Lithuanian Animal Ethics Committee.

### Experimental animals

Lithuanian White pigs were fasted 12 hours before the experiment but were allowed free access to water. All pigs were premedicated with intramuscular ketamine hydrochloride (20 mg/kg) and xylazine (2 mg/kg), followed by cannulation of an ear vein. Orotracheal intubation (6.5- to 7.5-mm tracheal tubes) was performed after intravenous induction of general anesthesia with 6 mg/kg of thiopental sodium. The animals were ventilated with a volume-controlled mode with a positive end-expiratory pressure of 5 cm H_2_O (Drager, Lubeck, Germany). Tidal volume was kept at 10 to 12 ml/kg, and the respiratory rate was adjusted (14 to 16 breaths/min) to maintain end-tidal carbon dioxide tension between 35 and 45 mm Hg (4.7 to 6.0 kPa). General anesthesia and muscle relaxation were maintained with a continuous intravenous infusion of fentanyl, 15 μg/kg/h, midazolam (0.2 mg/kg/h), and pipecuronium bromide IV boluses as needed. The stomach was emptied with an orogastric tube.

Core temperature was monitored via the pulmonary artery catheter and kept > 38.0°C, by using warmed solutions and heating mattresses. To maintain arterial blood glucose levels between 4.5 and 7 m*M*, 10% glucose was infused during the experiment.

### Surgical procedures

Both femoral veins and the right femoral artery were surgically dissected and catheterized. An arterial catheter was placed into the right femoral artery to measure invasive arterial blood pressure and to obtain blood gases. A standard thermodilution 7F Swan-Ganz catheter (TD-I; B. Braun Medical, Bethlehem, PA, USA) was advanced into the pulmonary artery through an introducer in the right femoral vein. A central vein catheter was introduced into the left femoral vein for delivery of medications and fluids. A Foley catheter was inserted into the urinary bladder through a small suprapubic incision.

A proximal-loop jejunostomy was constructed for microcirculatory assessment through a 5- to 7-cm incision on the right side of the abdominal wall. The jejunostomy was covered with moisturized gauze with local hourly administration of saline solution to maintain mucosal integrity.

Ringer lactate solution was infused at rate of 5 ml/kg/h during the surgical procedures. After surgical preparations, the animals were allowed to stabilize for 1 hour before recording the baseline measurements.

### Protocol and measurements

After basal measurements, the pigs were assigned randomly to either the septic or the sham group. Sepsis was induced by an infusion of live *Escherichia coli (E. coli*, 1.8 × 10^9^/kg colony-forming units) over a 1-hour period. *E. coli *was isolated from blood cultures from a patient who died of septic shock, according to a previous published model [10]. During and after the infusion of *E. coli*, Ringer lactate was continuously titrated to maintain CVP and PAOP at baseline levels. In addition to this, mean arterial pressure (MAP) was maintained > 70 mm Hg by providing standardized fluid boluses of 250 ml in 15 minutes until the increase in cardiac index (CI) did not exceed 10%.

Measurements, including systemic, pulmonary arterial, right atrial, PAOP, and cardiac output measured with the thermodilution technique, were registered at baseline and every hour until 5 hours after the start of the *E. coli*. Derived hemodynamic variables were calculated according to the usual formulas and indexed for body surface area. Arterial blood samples were obtained at baseline and after 5 hours for measurement of hemoglobin, hematocrit, potassium, arterial lactate, and arterial blood gases (ABL 500; Radiometer, Copenhagen, Denmark).

Microcirculatory measurements were performed at baseline and at 3 and 5 hours after the start of live *E. coli *infusion. After 5 hours of experiment, pigs were killed with a bolus injection of thiopental sodium and KCl.

### Videomicroscopic measurements and analysis

Images of the sublingual, conjunctival, jejunal, and rectal microcirculation were obtained with SDF videomicroscopy (Microscan; Microvision Medical, Amsterdam, The Netherlands) [11]. After gentle removal of saliva and other secretions with isotonic saline-drenched gauze, the device was applied to the sublingual region, avoiding pressure artifacts by establishing a threshold image. Before evaluation of conjunctiva, the eye was washed with 0.9% saline solution. An SDF objective with a disposable sterile lens cap was gently applied to the perilimbal surface of the bulbar conjunctiva, preventing the collapse and decrease of flow of large vessels. Movement artifacts were prevented by attaching the SDF objective to a finger of the other hand placed on the periocular bones and by additional fixation of the eyelid. An additional person was often needed to optimize the brightness and sharpness of the image. The eye conjunctiva was regularly rinsed with 0.9% saline solution to prevent local inflammation and was further protected by wet gauze on the eye between measurements. Measurements were performed on the same eye during all experiments.

The microcirculation of the small intestine was investigated through a jejunostomy by insertion of an SDF objective to the depth of 5 to 7 cm from the edge of the stoma and slight angulation.

The microcirculation of rectum was investigated through the anus by insertion of an SDF objective to the depth of 6 to 10 cm from the edge.

Sequences of 20 seconds from at least three areas within one vascular bed were recorded on a hard disk of a personal computer and AVA v3.0 software (Microvision Medical, Amsterdam, The Netherlands). Video clips were blindly analyzed offline by two investigators in random order to prevent coupling. Assessment of microcirculatory parameters of convective oxygen transport (microvascular flow index (MFI) and proportion of perfused vessels (PPVs)), and diffusion distance (perfused vessel density (PVD) and total vessel density (TVD)) was done according to published recommendations [12]. Vessels were separated into large (mostly venules) and small (mostly capillaries) by using a diameter cutoff value of 20 μm. The final MFI score is the average value of all quadrants in three different areas of one vascular bed. The heterogeneity index was calculated as the difference between the highest and lowest MFI, divided by the mean MFI of all sites at a single time point [5]. Microvascular beds of gut villi (small intestine) and crypts (colon) consist only of small vessels. In addition to MFI, we also calculated PPV as the percentage of perfused villi or crypts divided by the total number of villi or crypts times 100%, in accordance with definitions in the sublingual region [12].

### Statistical analysis

The primary end point was the MFI of small vessels in all investigated locations. The secondary end point was the correlation between sublingual and others locations of the microcirculation. The Statistical Package for Social Sciences (SPSS 15.1 for Windows, Chicago, IL, USA) was used for statistical analysis. Data were assessed for normality by using the Shapiro-Wilk test and presented as mean ± SD. Repeated-measures analysis of variance (ANOVA) for time comparison was used with Bonferroni correction for *post hoc *analysis. Differences between sepsis and control animals were analyzed by using the Mann-Whitney *U *test. Correlation between the sublingual microcirculation and that of other locations was assessed with the Spearman rho. A *P *value < 0.05 was considered statistically significant.

## Results

In total, 17 pigs were randomly assigned to the sepsis (*n *= 13; 27.5 ± 6.2 kg) or sham (*n *= 4; 30.0 ± 5.5 kg) group. Four pigs died within the first 15 minutes after the beginning of the *E. coli *infusion and were excluded from further analysis. All the other animals in the sepsis group (*n *= 9) developed a hypodynamic septic condition, characterized by the low cardiac output, increased systemic vascular resistance, and increased pressure in the pulmonary artery (Table 1). At least one episode of systemic arterial hypotension within the first 2 hours (54 ± 45 minutes) after starting the infusion of *E. coli *was recorded in all sepsis cases. These episodes of decreased arterial blood pressure were managed with infusion of crystalloid solutions, according to protocol. The lactate concentration in the arterial blood of septic animals increased statistically significantly compared with that of the control group (Table 2). None of the animals had hypoxemia.

**Table 1:** Systemic hemodynamic variables over time in septic (*n *= 9) and sham (*n *= 4) groups

Variables	Group	Baseline	3 hours	5 hours	ANOVA (*P*)
HR, beats/min	Sepsis	85 ± 18	104 ± 34	101 ± 25	0.368
	Sham	84 ± 8	99 ± 12	98 ± 4	0.400
MAP, mm Hg	Sepsis	99 ± 17	80 ± 25	92 ± 29	0.417
	Sham	107 ± 18	113 ± 4	92 ± 5	0.607
MPAP, mm Hg	Sepsis	19 ± 6	36 ± 7^a^	37 ± 6^a^	0.022
	Sham	23 ± 9	21 ± 2	20 ± 5	0.867
CI, L/min/m^2^	Sepsis	3.6 ± 0.8	2.4 ± 1.1^a^	1.8 ± 0.6^a^	0.028
	Sham	3.4 ± 0.1	4.2 ± 0.1	4.7 ± 2.0	0.368
SVRI, dynes/sec/cm^3^	Sepsis	1,938 ± 350	3,304 ± 1,513^a^	4,379 ± 1,640^a^	0.007
	Sham	2,065 ± 12	,2081 ± 128	1,776 ± 404	0.607
CVP, mm Hg	Sepsis	6 ± 2	8 ± 3	7 ± 3	0.300
	Sham	5 ± 1	4 ± 1	3 ± 1	0.215
PAOP, mm Hg	Sepsis	9 ± 4	25 ± 10^a^	24 ± 14^a^	0.021
	Sham	6 ± 1	14 ± 8	11 ± 7	0.313
Urine output, ml/h	Sepsis	89 ± 48	118 ± 80	71 ± 40	0.147
	Sham	116 ± 70	175 ± 60	150 ± 70	0.156
Cumulative amount of fluids, ml	Sepsis	1,100 ± 390	3,180 ± 834	4,425 ± 1,160	-
	Sham	1,250 ± 350	3,370 ± 450	4,670 ± 880	-

CI, cardiac index; CVP, central venous pressure; HR, heart rate; MAP, mean arterial pressure; MPAP, mean pulmonary artery pressure; PAOP, pulmonary artery occlusion pressure; SVRI, systemic vascular resistance index. Data are expressed as mean ± SD. ^a^*P *< 0.05 versus baseline.

**Table 2:** Blood gases and metabolic variables in septic (*n *= 9) and sham (*n *= 4) animals

Variables	Group	Baseline	5 hours
P_a_O_2_, mm Hg	Sepsis	150 ± 16	114 ± 35^a^
	Sham	130 ± 39	113 ± 24
P_a_CO_2_, mm Hg	Sepsis	32 ± 3	37 ± 9
	Sham	36 ± 4	37 ± 4
Arterial pH	Sepsis	7.5 ± 0.03	7.32 ± 0.10^a^
	Sham	7.49 ± 0.01	7.45 ± 0.06
HCO_3_, m*M*	Sepsis	26.6 ± 0.7	21.1 ± 3.7^a^
	Sham	27.6 ± 0.8	26.7 ± 0.1
Lactate, m*M*	Sepsis	0.8 ± 0.2	6.4 ± 2.3^a^
	Sham	1.1 ± 0.2	1.1 ± 0.2
Hematocrit, %	Sepsis	30 ± 3	32 ± 4
	Sham	29 ± 1	29 ± 1
Temperature,°C	Sepsis	38.3 ± 0.4	38.9 ± 0.9
	Sham	39.2 ± 1.1	38.8 ± 1.0

Data are expressed as mean ± SD. ^a^*P *< 0.05 versus baseline.

### Evaluation of microcirculation

**Figure 1:**
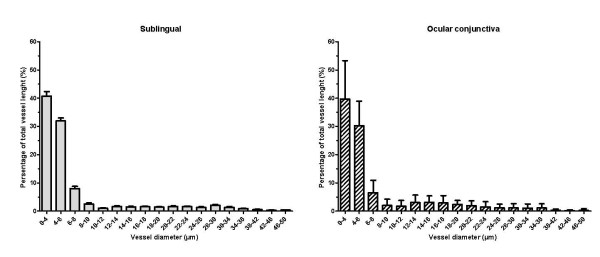
**Distribution of vessels in pig ocular conjunctiva (*n *= 39 images) and sublingual tissue (*n *= 39 images) according to vessel diameter and length**.

The kappa coefficient for interrater variability was 0.86 (*P *< 0.001), and for intrarater variability was 0.79 (*P *< 0.001). The percentage of disagreement for the MFI in the various vascular beds was as follows: eye, 5.6%; sublingual, 5.2%; intestinal stoma, 8.6%; and rectal, 9.0%. Initial microcirculatory parameters at the starting point were similar in sepsis and control groups (Table 3). The distribution of vessels according to internal diameter and length in sublingual and eye conjunctival locations at baseline are shown in Figure 1. In the conjunctiva, 77% of the vessel length was < 8 μm in internal diameter, and 92% was < 20 μm. In the sublingual mucosa, 81% of the vessel length was < 8 μm in internal diameter, and 90% was < 20 μm.

**Table 3:** Microcirculatory variables over time in septic (*n *= 9) and sham (*n *= 4) animals

	Group	Baseline	3 hours	5 hours	ANOVA (*P*)
Sublingual mucosa					
MFI s	Sepsis	2.73 ± 0.24	1.56 ± 0.30^a^	1.54 ± 0.34^a^	0.001
	Sham	2.89 ± 0.19	3.00 ± 0.00	3.00 ± 0.00	0.368
MFI l	Sepsis	3.00 ± 0.00	2.94 ± 0.19	3.00 ± 0.00	0.368
	Sham	3.00 ± 0.00	3.00 ± 0.00	3.00 ± 0.00	0.999
PPV s, %	Sepsis	91.4 ± 3.3	71.0 ± 8.1^a^	70.2 ± 8.4^a^	0.011
	Sham	95.0 ± 0.3	93.8 ± 1.9	94.3 ± 2.0	0.264
PVD s, 1/mm	Sepsis	14.5 ± 3.2	11.6 ± 2.7	12.4 ± 1.9	0.197
	Sham	15.3 ± 1.8	15.4 ± 2.3	16.5 ± 2.0	0.100
TVD s, mm/mm^2^	Sepsis	27.9 ± 2.4	27.8 ± 2.8	30.0 ± 3.6	0.197
	Sham	29.9 ± 3.9	30.7 ± 2.8	32.6 ± 5.0	0.529
TVD l, mm/mm^2^	Sepsis	6.0 ± 1.3	7.1 ± 1.1	7.3 ± 2.0	0.197
	Sham	8.4 ± 1.4	8.2 ± 1.3	8.1 ± 3.3	0.761
Het MFI	Sepsis	0.17 ± 0.14	0.44 ± 0.40	0.37 ± 0.24	0.250
	Sham	0.06 ± 0.11	0.00 ± 0.00	0.00 ± 0.00	0.368
Ocular conjunctiva					
MFI s	Sepsis	2.74 ± 0.21	2.14 ± 0.33^a^	2.00 ± 0.52^a^	0.004
	Sham	2.94 ± 0.10	2.83 ± 0.17	2.83 ± 0.29	0.867
MFI l	Sepsis	3.00 ± 0.00	2.98 ± 0.04	3.00 ± 0.00	0.135
	Sham	2.94 ± 0.10	2.94 ± 0.10	3.00 ± 0.00	0.368
PPV s, %	Sepsis	93.4 ± 3.1	84.4 ± 4.2^a^	78.4 ± 8.7^a^	0.009
	Sham	95.5 ± 0.3	94.4 ± 1.6	94.5 ± 1.2	0.441
PVD s, 1/mm	Sepsis	15.7 ± 3.0	12.7 ± 1.8^a^	11.7 ± 3.6	0.042
	Sham	16.9 ± 3.0	16.3 ± 3.6	15.2 ± 3.8	0.160
TVD s, mm/mm^2^	Sepsis	29.5 ± 4.8	26.6 ± 3.9	25.9 ± 8.4	0.135
	Sham	29.4 ± 6.3	29.1 ± 6.6	28.1 ± 7.8	0.761
TVD l, mm/mm^2^	Sepsis	7.9 ± 2.0	7.4 ± 2.3	8.0 ± 1.5	0.846
	Sham	8.6 ± 3.4	6.8 ± 1.8	8.5 ± 4.8	0.529
Het MFI	Sepsis	0.13 ± 0.12	0.38 ± 0.32	0.36 ± 0.31	0.113
	Sham	0.06 ± 0.10	0.09 ± 0.09	0.10 ± 0.17	0.867
Jejunal mucosa					
MFI s	Sepsis	2.89 ± 0.13	1.90 ± 0.32^a^	2.10 ± 0.43^a^	< 0.0001
	Sham	3.00 ± 0.00	2.67 ± 0.14	2.64 ± 0.32	0.124
PPV v, %	Sepsis	95.2 ± 2.8	82.6 ± 6.4^a^	84.3 ± 8.8^a^	0.022
	Sham	99.2 ± 1.4	92.0 ± 7.1	92.6 ± 1.7	0.368
MFI Het	Sepsis	0.11 ± 0.11	0.37 ± 0.19^a^	0.50 ± 0.27^a^	< 0.0001
	Sham	0.00 ± 0.00	0.09 ± 0.01	0.10 ± 0.10	0.178
Rectal mucosa					
MFI s	Sepsis	2.74 ± 0.15	1.95 ± 0.28^a^	1.83 ± 0.19^a^	< 0.0001
	Sham	3.00 ± 0.00	2.92 ± 0.12	2.67 ± 0.12	0.156
PPV c	Sepsis	93.0 ± 1.5	78.6 ± 6.9^a^	80.6 ± 6.3^a^	0.018
	Sham	97.1 ± 1.0	95.4 ± 1.6	93.1 ± 0.4	0.156
Het MFI	Sepsis	0.05 ± 0.08	0.36 ± 0.20^a^	0.21 ± 0.14^a^	0.015
	Sham	0.00 ± 0.00	0.09 ± 0.12	0.28 ± 0.01	0.156

Het MFI, coefficient of MFI s variation; MFI s, microvascular flow index of small vessels; MFI l, microvascular flow index of large vessels; PPVc, percentage of perfused crypts; PPVs, percentage of perfused small vessels; PPVv, percentage of perfused villi; PVDs, perfused vessel density of small vessels; TVD s, total vessel density of small vessels. Data are expressed as mean ± SD. ^a^*P *< 0.05 versus baseline.

**Figure 2:**
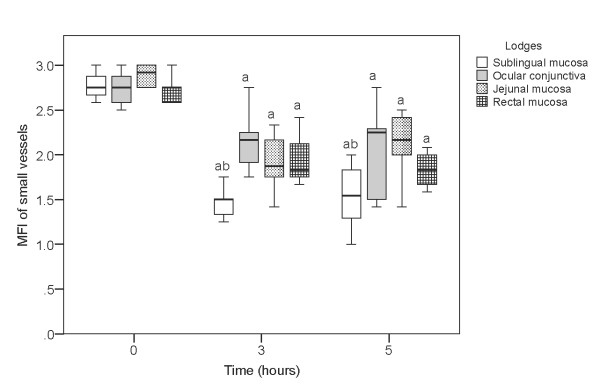
**Boxplots of microvascular flow index (MFI) of small vessels during 5 hours of experiment in the sepsis group**. **(a) ***P *< 0.05 versus baseline; **(b) ***P *< 0.05 versus other locations.

A significant decrease of microcirculatory parameters of convective oxygen transport (MFI and PPV of small vessels) was observed in all locations (sublingual, jejunal, rectal mucosa, and ocular conjunctiva) in the sepsis group (Table 3). In contrast, we observed a significant decrease in only one parameter of diffusion distance (PVD of small vessels) in the conjunctival but not in the other locations. TVD remained unaltered in both the sublingual and conjunctival microcirculatory beds (Table 3). Statistical analysis of MFIs in different microcirculatory locations of the sepsis animals revealed that the lowest MFIs could be detected in the sublingual region 3 hours and 5 hours after the induction of sepsis with infusion of *E. coli *(Figure 2).

We observed no correlation between MFI and CI, MAP, or lactate in almost all locations. Only sublingual MFI significantly correlated with lactate (*r_s _*= 0.9; *P *= 0.037).

### Association between sublingual microcirculation and other locations

**Figure 3:**
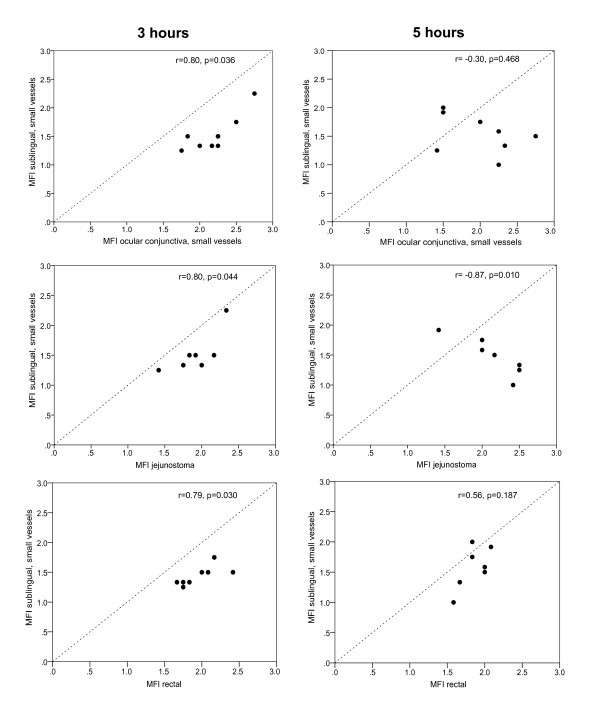
**Scatters of microvascular flow index (MFI) of small vessels in the sublingual region versus the MFI in the ocular conjunctiva, jejunal stoma, and rectum**. Left-sided scatters represent 3 hours of experiment; right sided, 5 hours of experiment.

A statistically significant correlation could be demonstrated between the sublingual and conjunctival (*r_s _*= 0.80; *P *= 0.036), sublingual and jejunal (*r_s _*= 0.80; *P *= 0.044), and sublingual and rectal (*r_s _*= 0.79; *P *= 0.030) MFI measurements 3 hours after the start of *E. coli *infusion. However, this correlation between the sublingual and conjunctival, and the jejunal and rectal regions disappeared 5 hours after the start of *E. coli *infusion (Figure 3). Correlations of MFI between others areas except the sublingual are shown in Additional file 1. Correlations between beds at different times for PPV, PVD, and TVD of small vessels are shown in Additional file 2.

**Figure 4:**
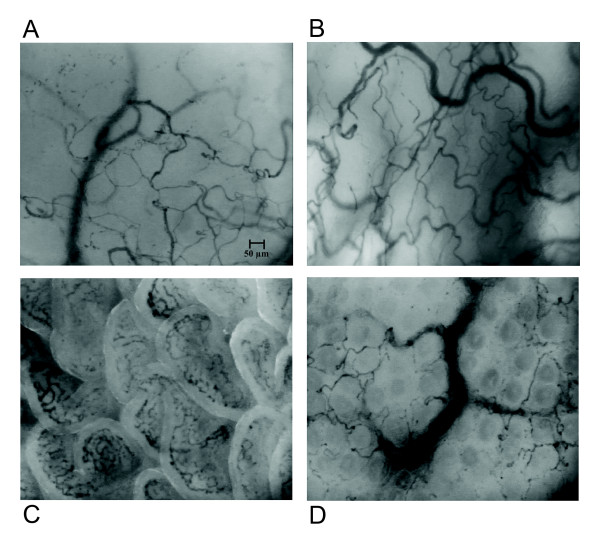
**Digital microphotographs of microcirculation at 3 hours in sepsis group**. **(A) **Sublingual mucosa; **(B) **ocular conjunctiva; **(C) **jejunal mucosa; **(D) **rectal mucosa.

We observed a tendency toward an increased heterogeneity index level at 3 hours after the start of *E. coli *infusion (0.11 ± 0.07 versus 0.25 ± 0.15; *P *= 0.068), and a significant difference at 5 hours after the start of *E. coli *infusion (0.11 ± 0.07 versus 0.59 ± 0.20; *P *= 0.025) in comparison with baseline. Digital microphotographs of microcirculation in the sepsis group are presented in Figure 4.

## Discussion

In this early hypodynamic sepsis model, alterations of microcirculation were observed in four locations (sublingual, conjunctival, jejunal, and rectal mucosa) during various time points with SDF videomicroscopy. We established a time-dependent correlation of MFI between the sublingual and the other three locations, such as eye conjunctival, jejunal, and rectal mucosa. After 3 hours, a correlation was detected, but after 5 hours, it disappeared. Previous animal data obtained with laser Doppler flowmetry show that the distribution of blood flow in the microcirculatory beds of splanchnic organs (gastric, jejunal, and colon mucosa, liver, and pancreas) is heterogeneous, both in early hypodynamic and in hyperdynamic septic shock [8]. Boerma and colleagues [7] showed, in a clinical study using OPS imaging, that on day 1 of abdominal sepsis, a complete dispersion of flow occurs between sublingual and intestinal microcirculation, but on day 3, the correlation appeared to be restored. Some authors criticized this study because of the possibility of increased intraabdominal pressure or local inflammatory factors.

Verdant and colleagues [13] published an experimental study using a hyperdynamic model of sepsis with *E. coli *instillation into the common bile duct in pigs. They demonstrated a significant association of PPV between sublingual and jejunal mucosa during 12 hours of the experiment.

We used an intravenous model of sepsis to minimize the impact of local factors on microcirculation and observed the time-dependent dispersion of microcirculatory alterations also outside the abdomen. However, our results represent a different model with respect to the observed hypodynamic state of sepsis, which may represent differences either in volume status or in myocardial contractility. Furthermore, our observational time was shorter and was limited to 5 hours only. Nevertheless, the duration of the experiment reflects the time needed for stabilization of patients in clinical practice. Studies have shown that adequately resuscitated septic-shock patients typically exhibit a persistent hyperdynamic state with high cardiac output and low systemic vascular resistance [14]. A hypodynamic state of sepsis in humans can occur in cases of hypovolemia or cardiac depression due to cytokines (tumor necrosis factor (TNF)-α, interleukin (IL)-1β, IL-6, and others) [15]. Thus, our model of hypodynamic sepsis creates an early extreme condition with significantly altered microcirculation despite fluid administration.

We cannot rule out that the mentioned differences in sepsis models and time points for evaluation of microcirculation resulted in the differences of microcirculatory perfusion, when compared with those of previously published studies. In our study, loss of correlation is accompanied by a trend to an increase in the heterogeneity index.

We have not found any literature reports demonstrating correlation of microcirculatory alterations in the sublingual mucosa and the eye. The disappearance of correlation between sublingual and conjunctival microcirculation 5 hours after induction of sepsis could be explained by a difference in organ-specific response to inflammation, with possible septic brain damage [16].

In our experiment, the most pronounced alterations of flow (MFI) were observed in the sublingual part during the complete experiment. This indicates that the monitoring of MFI in the sublingual region can help in the identification of any alterations in microcirculation in others locations, when local factors are not applied.

Microcirculatory variables in observed locations were not related to MAP or CI. These additional clinical data demonstrated that microcirculatory alterations occur independent of systemic hemodynamic changes during sepsis [17-19]. It is of note, however, that the observations were done within a limited range of cardiac output and blood pressures.

Our study has certain limitations, including differences in human and pig physiology, diminishing the ability to translate the obtained data directly into clinical practice. For example, excessive release of cytokines, such as TNF-α, in pigs is known to induce pulmonary hypertension and cardiac depression [20]. Conversely, we did not give any vasopressors and antibiotics during our experiments (which would be the case in a clinical setting), as these factors potentially affect the microcirculation. We did not measure intraabdominal and intracranial pressure during our study. Also we cannot exclude that surgical stoma preparation may have influenced the intestinal microcirculation. Although the stoma was prepared by a professional surgeon, differences between control and sepsis animals during the experiment were significant.

## Conclusions

Microcirculatory alterations were observed in all investigated locations, including sublingual, jejunal, and rectal mucosa, and conjunctiva of the eye at the same time points during experimental sepsis. The most pronounced alterations of microcirculation during experiment were observed in the sublingual area. A time-dependent correlation exists between sublingual and other evaluated locations of the microcirculation during the course of a hypodynamic state of sepsis.

## Key messages

• Microcirculatory alterations due decreased microvascular flow index of small vessels and proportion of perfused capillaries were observed at the same time point in sublingual mucosa, conjunctiva of the eye, and jejunal and rectal mucosa during sepsis.

• A time-dependent direct association of microcirculation exists between sublingual and conjunctival, and jejunal and rectal mucosa during the course of a hypodynamic state of sepsis.

## Abbreviations

ANOVA: analysis of variance; CI: cardiac index; CVP: central venous pressure; *E. coli: Escherichia coli*; MAP: mean arterial pressure; OPS: orthogonal polarization spectral; PAOP: pulmonary artery occluded pressure; MFI: microvascular flow index; PPV: proportion of perfused vessels; PVD: perfused vessel density; SDF: sidestream dark field; TVD: total vessel density.

## Competing interests

The authors state that they have no conflicts of interest.

## Authors' contributions

AP conceived the study, performed SDF imaging, and wrote the first draft; AP, VV, RP, and ZD developed the animal model for microcirculation evaluation; AP and RR analyzed SDF images; VP, DV, and PD participated in the design of the study; and CB performed statistical analysis and wrote the final manuscript. All authors read and approved the final manuscript.

## Supplementary Material

Additional file 1 **Correlations of microvascular flow index (MFI) between conjunctival and jejunal, conjunctival and rectal, rectal and jejunal areas at 3 hours and 5 hours in sepsis group**. An additional scatterplot file shows correlations of MFI between other areas except sublingual. Left-sided scatters represent 3 hours of experiment; right-sided, 5 hours of experiment. Click here for file

Additional file 2 **Correlations between microcirculatory beds at 3 hours and 5 hours in the sepsis group**. An additional text file shows correlations between beds at different times for the proportion of perfused vessels (PPVs), perfused vessel density (PVD), and total vessel density (TVD) of small vessels. Click here for file

## References

[B1] Ince C The microcirculation is the motor of sepsis Crit Care 2005 9 Suppl 4 S13 S19 10.1186/cc3753 16168069 PMC3226164

[B2] Vincent JL Rello J Marshall J Silva E Anzueto A Martin CD Moreno R Lipman J Gomersall C Sakr Y Reinhart K EPIC II Group of Investigators International study of the prevalence and outcomes of infection in intensive care units JAMA 2009 302 2323 2329 10.1001/jama.2009.1754 19952319

[B3] Rivers E Nguyen B Havstad S Ressler J Muzzin A Knoblich B Peterson E Tomlanovich M the Early Goal-Directed Therapy Collaborative Group Early goal-directed therapy in the treatment of severe sepsis and septic shock N Engl J Med 2001 345 1368 1377 10.1056/NEJMoa010307 11794169

[B4] De Backer D Creteur J Preiser JC Dubois MJ Vincent JL Microvascular blood flow is altered in patients with sepsis Am J Respir Crit Care Med 2002 166 98 104 10.1164/rccm.200109-016OC 12091178

[B5] Trzeciak S Dellinger RP Parrillo JE Guglielmi M Bajaj J Abate NL Arnold RC Colilla S Zanotti S Hollenberg SM Early microcirculatory perfusion derangements in patients with severe sepsis and septic shock: relationship to hemodynamics, oxygen transport, and survival Ann Emerg Med 2007 49 88 98 10.1016/j.annemergmed.2006.08.021 17095120

[B6] Spanos A Jhanji S Vivian-Smith A Harris T Pearse RM Early microvascular changes in sepsis and severe sepsis Shock 2010 33 387 391 10.1097/SHK.0b013e3181c6be04 19851124

[B7] Boerma EC van der Voort PH Spronk PE Ince C Relationship between sublingual and intestinal microcirculatory perfusion in patients with abdominal sepsis Crit Care Med 2007 35 1055 1060 10.1097/01.CCM.0000259527.89927.F9 17334238

[B8] Hiltebrand LB Krejci V Banic A Erni D Wheatley AM Sigurdsson GH Dynamic study of the distribution of microcirculatory blood flow in multiple splanchnic organs in septic shock Crit Care Med 2000 28 3233 3241 10.1097/00003246-200009000-00019 11008987

[B9] Schaser KD Settmacher U Puhl G Zhang L Mittlmeier T Stover JF Vollmar B Menger MD Neuhaus P Haas NP Noninvasive analysis of conjunctival microcirculation during carotid artery surgery reveals microvascular evidence of collateral compensation and stenosis-dependent adaptation J Vasc Surg 2003 37 789 797 10.1067/mva.2003.139 12663979

[B10] Mohr M Höpken U Oppermann M Mathes C Goldmann K Siever S Götze O Burchardi H Effects of anti-C5a monoclonal antibodies on oxygen use in a porcine model of severe sepsis Eur J Clin Invest 1998 28 227 234 10.1046/j.1365-2362.1998.00260.x 9568469

[B11] Goedhart P Khalilzada M Bezemer R Merza J Ince C Sidestream dark field (SDF) imaging: a novel stroboscopic LED ring-based imaging modality for clinical assessment of the microcirculation Opt Express 2007 15 15101 15114 10.1364/OE.15.015101 19550794

[B12] De Backer D Hollenberg S Boerma C Goedhart P Buchele G Ospina-Tascon G Dobbe I Ince C How to evaluate the microcirculation: report of a round table conference Crit Care 2007 11 R101 10.1186/cc6118 17845716 PMC2556744

[B13] Verdant CL De Backer D Bruhn A Clausi CM Su F Wang Z Rodriguez H Pries AR Vincent JL Evaluation of sublingual and gut mucosal microcirculation in sepsis: a quantitative analysis Crit Care Med 2009 37 2875 2881 10.1097/CCM.0b013e3181b029c1 19770750

[B14] Kumar A Haery C Parrillo JE Myocardial dysfunction in septic shock: Part I. Clinical manifestation of cardiovascular dysfunction J Cardiothorac Vasc Anesth 2001 15 364 376 10.1053/jcan.2001.22317 11426372

[B15] Reilly JM Cunnion RE Burch-Whitman C Parker MM Shelhamer JH Parrillo JE A circulating myocardial depressant substance is associated with cardiac dysfunction and peripheral hypoperfusion (lactic acidemia) in patients with septic shock Chest 1989 95 1072 1080 10.1378/chest.95.5.1072 2707065

[B16] Ebersoldt M Sharshar T Annane D Sepsis-associated delirium Intensive Care Med 2007 33 941 950 10.1007/s00134-007-0622-2 17410344

[B17] Dubin A Edul VS Pozo MO Murias G Canullan CM Martins EF Ferrara G Canales HS Laporte M Estenssoro E Ince C Persistent villi hypoperfusion explains intramucosal acidosis in sheep endotoxemia Crit Care Med 2008 36 535 542 10.1097/01.CCM.0000300083.74726.43 18216603

[B18] Taccone FS Su F Pierrakos C He X James S Dewitte O Vincent JL De Backer D Cerebral microcirculation is impaired during sepsis: an experimental study Crit Care 2010 14 R140 10.1186/cc9205 20667108 PMC2945121

[B19] De Backer D Creteur J Dubois MJ Sakr Y Koch M Verdant C Vincent JL The effects of dobutamine on microcirculatory alterations in patients with septic shock are independent of its systemic effects Crit Care Med 2006 34 403 408 10.1097/01.CCM.0000198107.61493.5A 16424721

[B20] Zanotti-Cavazzoni SL Goldfarb RD Animal models of sepsis Crit Care Clin 2009 25 703 719 vii 10.1016/j.ccc.2009.08.005 19892248

