# Supplementary figures and images for: Early course of microcirculatory perfusion in eye and digestive tract during hypodynamic sepsis
Source: Crit Care. 2012 May 15;16(3):R83. doi: 10.1186/cc11341 (PMC3580626; doi:10.1186/cc11341)

3 hours

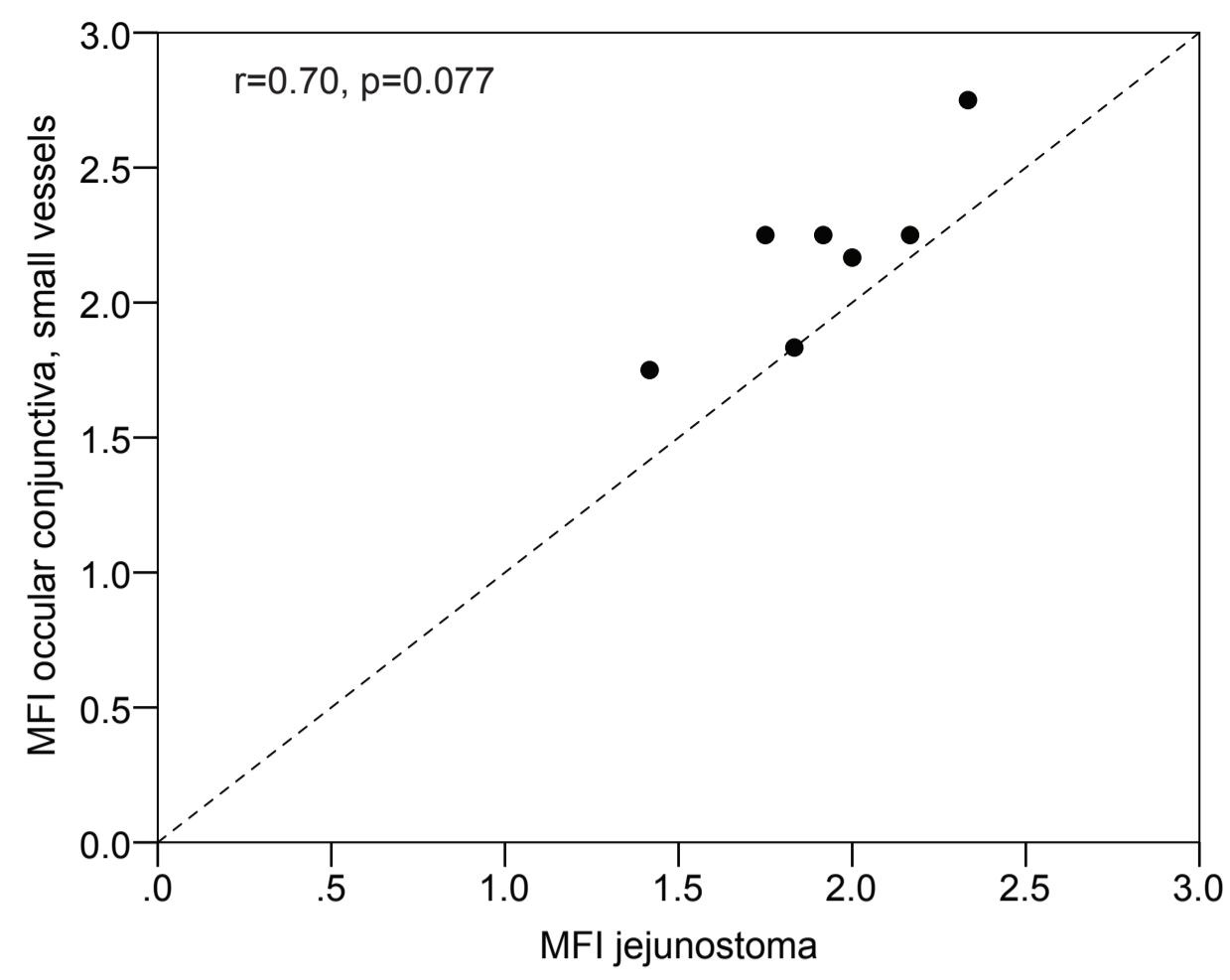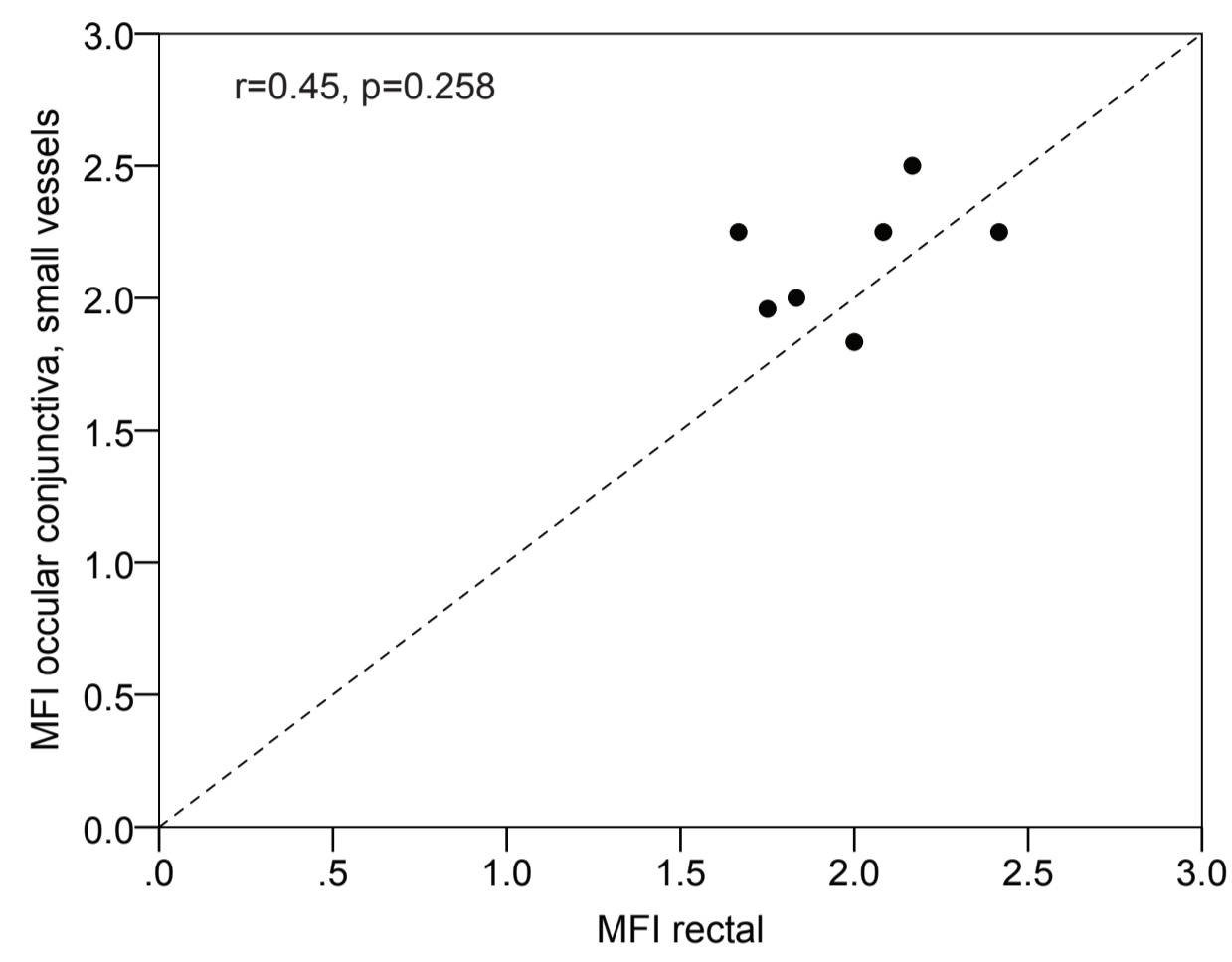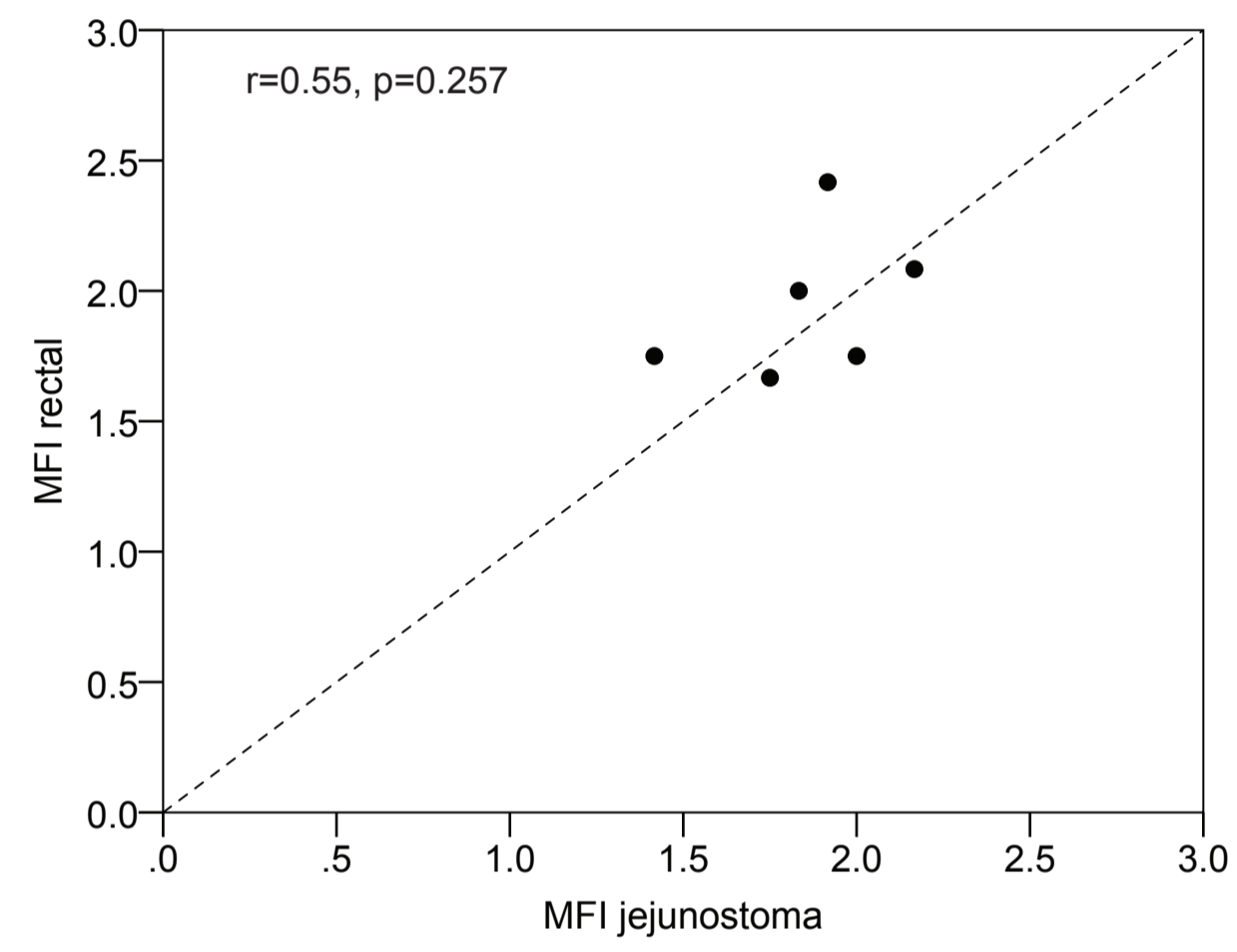

5 hours

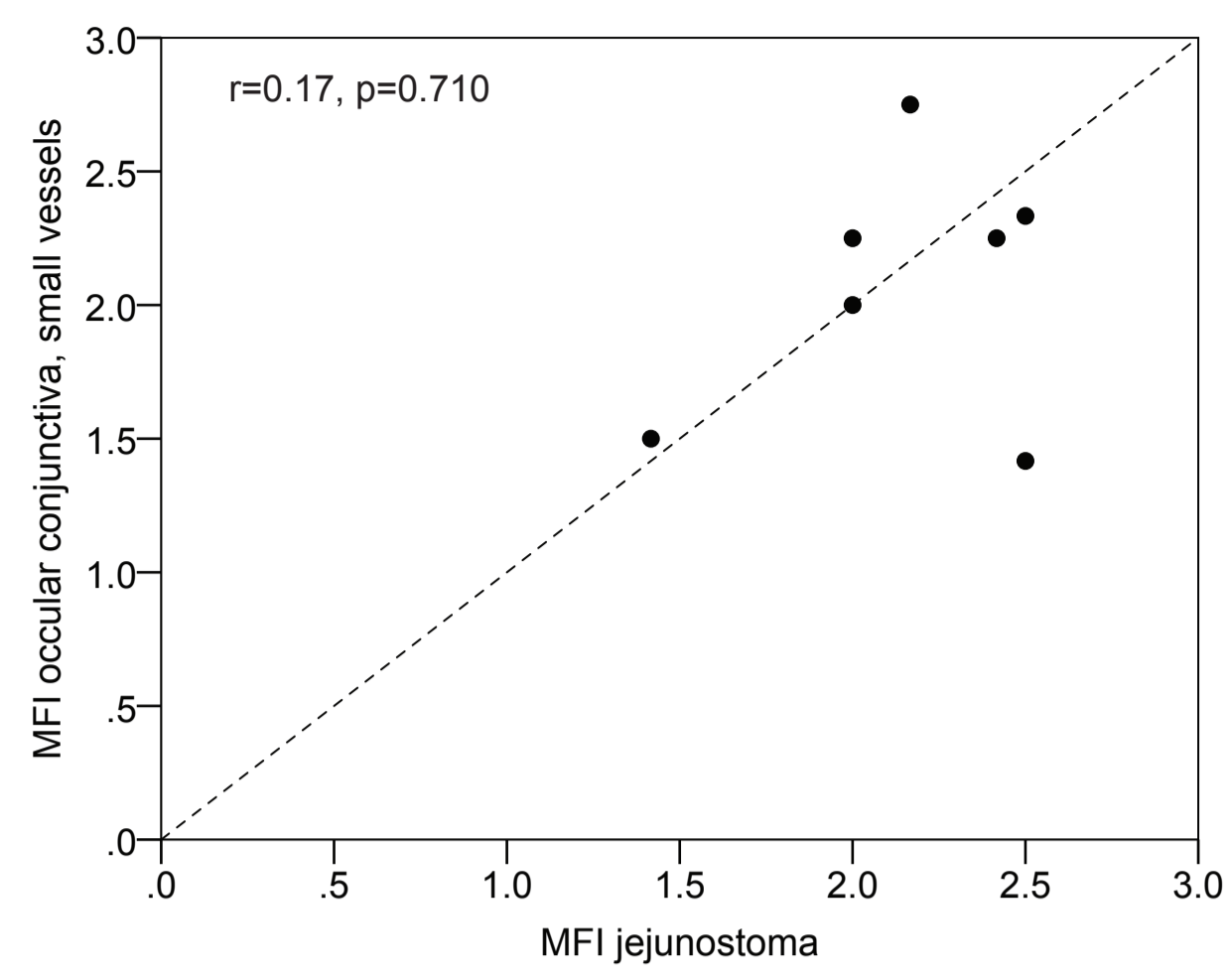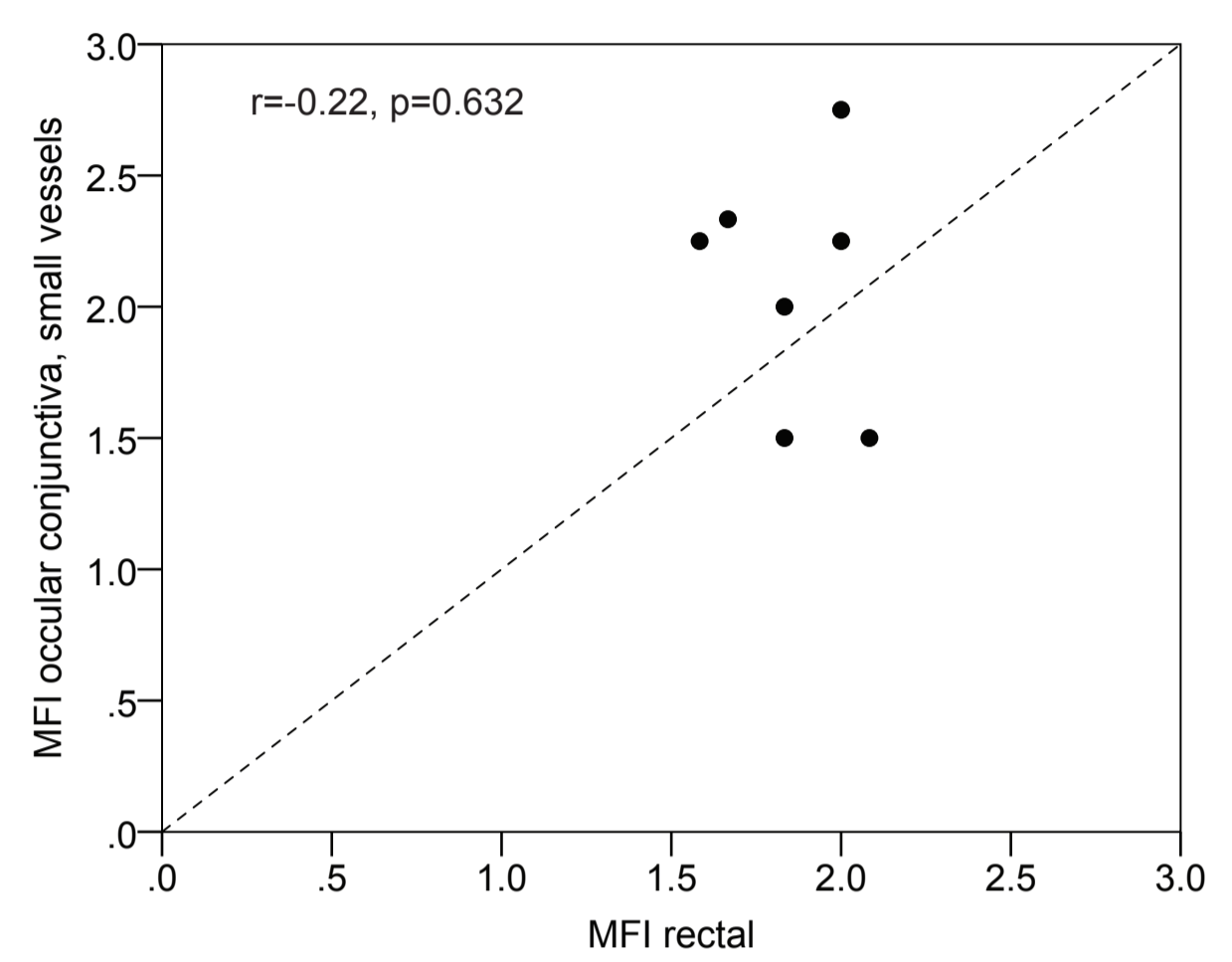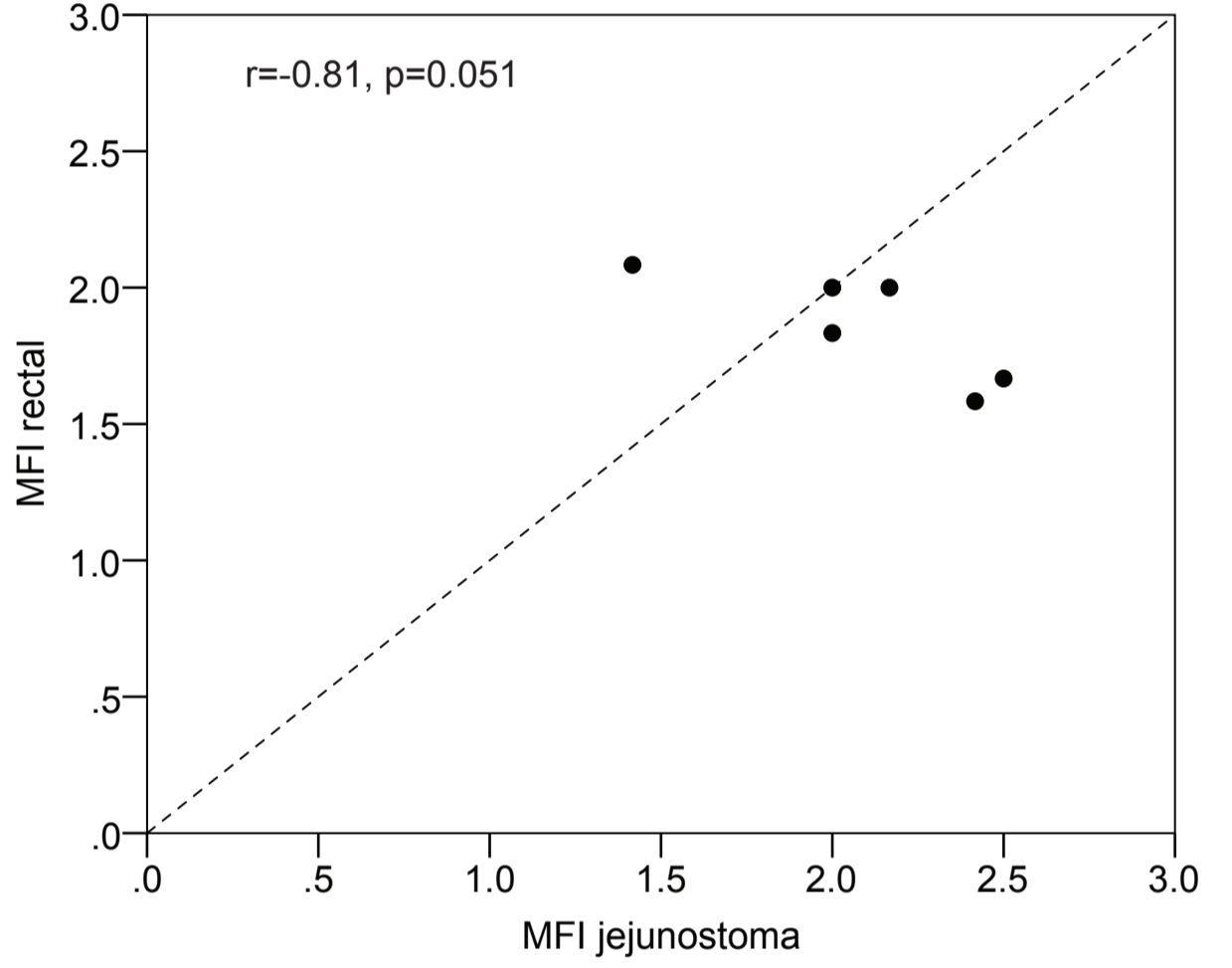

Supplement: Additional file 1 — Correlations of microvascular flow index (MFI) between conjunctival and jejunal, conjunctival and rectal, rectal and jejunal areas at 3 hours and 5 hours in sepsis group. An additional scatterplot file shows correlations of MFI between other areas except sublingual. Left-sided scatters represent 3 hours of experiment; right-sided, 5 hours of experiment. [file cc11341-S1.PDF]
