# Supplementary material for: Early course of microcirculatory perfusion in eye and digestive tract during hypodynamic sepsis
Source: Crit Care. 2012 May 15;16(3):R83. doi: 10.1186/cc11341 (PMC3580626; doi:10.1186/cc11341)
Supplement: Additional file 2 — Correlations between microcirculatory beds at 3 hours and 5 hours in the sepsis group. An additional text file shows correlations between beds at different times for the proportion of perfused vessels (PPVs), perfused vessel density (PVD), and total vessel density (TVD) of small vessels. [file cc11341-S2.RTF]

Additional File 2
Correlations between microcirculatory beds at 3 hours and 5 hours in sepsis group


	3 hours	p	5 hours	p	
	rs		rs		
PPV of small vessels					
Sublingual and conjunctival	0.72	0.030	-0.21	0.645	
Sublingual and jejunal	0.75	0.052	-0.50	0.391	
Sublingual and rectal	0.10	0.840	0.77	0.072	
Conjunctival and jejunal	0.57	0.180	0.40	0.600	
Conjunctival and rectal	0.22	0.641	-0.37	0.468	
Jejunal and rectal	0.40	0.500	-0.50	0.667	
PVD of small vessels					
Sublingual and conjunctival	0.50	0.253	-0.29	0.535	
TVD of small vessels
					
Sublingual and conjunctival	0.64	0.119	-0.50	0.253	
PPV - proportion of perfused vessels; PVD - perfused vessel density; TVD – total vessel density; rs – Spearman's correlation coefficient
